# Comparison in Adherence to Treatment between Patients with Mild–Moderate and Severe Reflux Esophagitis: A Prospective Study

**DOI:** 10.3390/jcm11113196

**Published:** 2022-06-03

**Authors:** Amir Mari, Wasef Na’amnih, Aiman Gahshan, Helal Saied Ahmad, Tawfik Khoury, Khitam Muhsen

**Affiliations:** 1 Gastroenterology Department, Nazareth Hospital, Azrieli Faculty of Medicine, Bar Ilan University, Ramat Gan 5290002, Israel; helal_sayid@yahoo.com (H.S.A.); tawfikkhoury1@hotmail.com (T.K.); 2 Department of Epidemiology and Preventive Medicine, School of Public Health, Sackler Faculty of Medicine, Tel Aviv University, Tel Aviv 6997801, Israel; wasef25@yahoo.com (W.N.); kmuhsen@tauex.tau.ac.il (K.M.); 3 Internal Medicine Department, Nazareth Hospital, Azrieli Faculty of Medicine, Bar Ilan University, Ramat Gan 5290002, Israel; aymanj9@hotmail.com

**Keywords:** reflux esophagitis, gastroscopy, adherence to treatment, diet, medications

## Abstract

Purpose: Gastro-esophageal reflux disease (GERD) is prevalent and causes erosive esophagitis (EE) with varying degrees of severity (A to D according to the Los Angeles Classification). Adherence to medical therapy is crucial for treatment success. We compared adherence to treatment recommendations between patients with EE grades C/D and A/B. Methods: A follow-up study was conducted during 2019–2020 among GERD patients who underwent a diagnostic gastroscopy 1–4 years earlier. Telephone interviews were conducted with patients diagnosed with severe EE grades C/D (n = 99) and randomly selected patients with mild–moderate EE grades A/B (n = 50). Patients with grades A/B were classified as adherent if they took proton pump inhibitors (PPIs) for 2–3 months as recommended. Patients with grades C/D were classified as adherent if they took medications for a prolonged period (>6 months) and performed a follow-up endoscopy as recommended. Results: The mean age of the participants was 44.6 years (SD = 15.1). The mean duration of PPIs therapy in patients with EE grades A/B was 9.4 months (SD = 8.7). Fourteen (14.2%) patients with EE grades A/B were non-adherent to treatment, compared to 21 (40.8%) patients with EE grades of C/D: adjusted OR = 0.06; CI 95% 0.02–0.18, *p* < 0.001. Follow-up endoscopy was performed by 44% of EE–C/D patients. Unmarried patients compared to married ones were less adherent (adjusted OR = 0.23; 95% CI 0.08–0.69, *p* < 0.001). Conclusions: Patients with esophagitis (EE–A/B) were more adherent to medical therapy when compared to patients with more severe esophagitis (EE–C/D).

## 1. Introduction

Gastro-esophageal reflux disease (GERD) is characterized by the regurgitation of gastroduodenal contents into the esophagus causing heartburn, chest pain, dysphagia, and other upper gastrointestinal symptoms [1,2]. The prevalence of GERD is increasing globally; the estimated prevalence ranges from 18.1 to 27.8% in North America and 8.8 to 25.9% in Europe [3]. The increase in GERD prevalence is probably related to changes in lifestyle and health behaviors such as smoking, diet and obesity [4].

The diagnosis reflux esophagitis is made by endoscopy that enables the grading of the disease severity. The spectrum of endoscopic findings in GERD ranges from non-erosive reflux disease (NERD) which is the most common, to erosive esophagitis (EE), passing through ulcers, stricture, premalignant lesions such as Barrette’s esophagus and esophageal carcinoma [5]. The Los Angeles scale is the most commonly used classification system for the grading of reflux esophagitis severity [6,7]. According to the Los Angeles scale there are for grades of esophagitis stages ranked from A to D, with D being the most severe disease [6].

Proton pump inhibitors (PPIs) are effective for the treatment of GERD that can result in endoscopic resolution of EE. PPI treatment was shown to be associated with a 70% improvement in esophagitis, 50% in NERD, and 30% in cases of functional heartburn [8,9,10]. According to the American College of Gastroenterology, the recommended treatment for patients with grade A and B esophagitis is a standard dose of a PPI once daily prescribed for 8 weeks [11]. A longer period of treatment or maintenance therapy with PPI for grade C and D esophagitis is generally needed since most patients will experience a recurrence of symptoms upon therapy discontinuation [11]. Moreover, a follow-up gastroscopy is recommended for patients diagnosed with grades C and D esophagitis after a course of PPI therapy to exclude Barrett’s esophagus [11].

Adherence to medical therapy is an important factor to control GERD symptoms and for the prevention of its complications. Poor adherence plays a key role in GERD management failure [12]. Generally, adherence is associated with many factors including, cultural, social, fear from chronic medication use and side effects, as well as factors related to patient such as awareness, understanding, level of education, as well as the financial state of the patient, a poor relationship between healthcare provider and patient, and unclear instructions and recommendations to the patient [13,14]. Notably, an accurate evaluation of non-adherence is challenging due to the lack of standardized in definition of adherence. The reported rates of non-adherence to medical treatment among GERD patients vary from 20 to 50% [15]. Nonetheless, to our knowledge, no comparative studies have assessed differences in adherence to medical treatment between patients with various degrees of erosive esophagitis.

In a previous study of 4148 patients with clinical diagnosis of GERD who underwent gastroscopy from 2014–2018 in northern Israel, 818 patients (19.7%) were diagnosed with grade A reflux esophagitis, 402 (9.6%) with grade B reflux esophagitis, and 72 (1.7%) and 16 (0.4%) patients, with C and D reflux esophagitis, respectively, while 2840 (60.0%) patients did not have endoscopic evidence of esophagitis^,^ and were diagnosed with NERD [16]. Patients with esophagitis grades A and B (mild-moderate) were released with the advice to take once daily PPI for 8–12 week and follow up if symptoms persist. Patients with grades C and D were recommended to take PPI as a long-term therapy and repeat gastroscopy after 8–12 weeks.

The aim of this study was to assess the adherence to treatment recommendations in patients with severe reflux esophagitis (grades C/D) when compared to patients with mild/moderate reflux esophagitis (grades A/B) and to assess potential correlates with adherence to recommendations.

## 2. Methods

### 2.1. Study Design and Population

A prospective study was undertaken among patients with GERD symptoms who underwent endoscopy at the Nazareth Hospital. The original cohort of the study included 4148 patients (52% females) aged 18 years or older; of those, 818 patients were diagnosed with EE grade A, 402 had grade B, and 72 and 16 patients had grades C and D esophagitis, respectively, while 2840 patients had NERD. The current study targeted all patients diagnosed with EE grade C or D and randomly selected patients with EE grades A/B, and it was conducted during 2019–2020, about 1–4 years following the baseline gastroscopy.

### 2.2. Endoscopy Procedure

The endoscopic procedures were performed by five senior specialists in gastroenterology who worked at the gastroenterology unit at Nazareth Hospital. The gastroenterologists were unaware to this ‘future’ study, therefore, no differential misclassification bias occurred.

### 2.3. Data Collection

Patients were contacted by phone and invited to take part in the study. Patients who agreed were interviewed to collect information on demographics and adherence to treatment recommendations. Information that was reported by the patients was validated against data on electronic medical files.

### 2.4. Definition of the Study Variables

The main dependent variable was adherence to treatment: Self-reports of the patients on the treatment (medications and follow-up examinations) of GERD they had used were taken between baseline and follow-up visit and whether they matched the recommendations was determined. The adherence assessment was based on well-structured clinical questions. In the framework of the current study, questions regarding adherence were produced in the Arabic and Hebrew languages, by researchers/experts in epidemiology and gastroenterology, speaking both languages at a native level. The translation went through several rounds between experts and the Arabic and Hebrew translations of the questions were tested between a separate set of researchers to assess face and content validity. The clarity of the questions after translation to Arabic and Hebrew was tested and discussed by experts until a consensus was reached. All translation and back translation was achieved prior to study initiation. A pilot study of 10 patients was completed before administering the study questionnaire to all participants to assess feasibility and acceptance.

Information on the names and dosage of the medications that the patients used as well as on the duration of treatment was collected. Adherence was defined based on the duration of PPI treatment. The recommendation for treatment duration is prolonged (i.e., >6 months) for grades C/D, and by 2–3 months for grades A/B. Patients with grades A/B were classified as adherent if they used PPIs for 2–3 months and non-adherent if the treatment duration was shorter than 2 months [11]. It is recommended that patients with EE grades C/D undergo a follow-up endoscopy after the initial diagnostic gastroscopy to monitor the development of Barret’s esophagus, therefore, this group’s incidence of undergoing the follow-up endoscopy was considered also in the classification of adherence. Patients with grades C/D were classified as adherent if they used PPIs for a prolonged period (>6 months) and performed follow-up endoscopy, otherwise they were classified as non-adherent. Information on PPI type (Lanton, Omepradex, Nexum or Controloc) and dosage consumed (20, 30, or 40 mg) was collected. This information was validated against data in medical records.

Moreover, we assessed the number of patients who followed the following recommendations/referrals: a change in dietary habits, a referral to gastroenterology clinic for follow-up, and a referral to a dietician clinic.

The main independent variable was the severity (grade) of EE: The severity (grades) of EE were determined by an experienced specialist in gastroenterology at baseline gastroscopy during 2014–2018 [16]; grades A and B were grouped as mild-to-moderate EE and grades C and D were grouped as severe EE.

To study the association between adherence and GERD symptoms severity, we have used the Reflux Disease Questionnaire (RDQ), a 12-item self-administered questionnaire, designed to assess the frequency and severity of heartburn, regurgitation, and dyspeptic complaints in the last week period [17]. The questionnaire is based on a Likert scale with responses/scores ranging from 0 to 5 for frequency and severity. Each subject’s score for each subclass of symptoms (heartburn, regurgitation, and dyspepsia) was calculated as the mean of item responses, with higher scores indicating more severe or frequent symptoms.

### 2.5. Potential Confounders

Age, sex, the number of schooling years, marital status, ethnicity, alcohol consumption, self-reported health status (the participant’s report on his/her health status as excellent, very good, good, not very good or bad), background illnesses (self-reports on diabetes, cardiovascular disease, stroke, cancer, kidney disease, organ transplant, hemodialysis, immunosuppressive therapies), and Charlson’s comorbidity index was calculated [18], as well as the incidence of hiatus Hernia, smoking (self-reports on being never, past or current smokers, and the number of cigarettes per day), physical activity (self-reports on being active almost daily, once-twice per week, once-twice per month, less than once per month or never), and current body mass index (BMI). BMI was calculated based on reported weight and height as weight (in kg)/higher (m)^2^.

### 2.6. Statistical Analysis

The participants’ demographics and clinical characteristics were described using mean and standard deviations (SD) for continuous variables and counts and percentages for categorical variables. The Chi-squared test was used to examine the association between adherence and the grade of esophagitis and other categorical variables, including the symptoms severity scores that were categorized by the median scores. The Fisher’s exact test was used when appropriate. The Student’s *t*-test was used for comparisons between the groups in continuous variables and the Mann–Whitney test was used for variables that did not follow a normal distribution. Logistic regression models, with adherence as the dependent variable and EE grade as the main independent variable were used to adjust for potential confounders (e.g., age, sex, ethnicity, and smoking and symptoms severity). Odds ratios (OR) and 95% confidence intervals (CIs) were obtained from these models. A *p* value < 0.05 was considered statistically significant. SPSS software version 25 was used in data analysis.

### 2.7. Sample Size Calculation

We assumed a power of 80%; type 1 error (2-sided test) 5%; 50% adherence in EE grade A/B and 75% in EE grade C/D; 2:1 ratio of A/B vs. C/D. Based on these assumptions, the minimum number of needed samples was 100 participants with EE A/B and 50 with EE C/D.

### 2.8. Ethical Aspects

The study protocol was approved by the Institutional Review Board (IRB) at the Nazareth hospital. Consent to participate in the study was obtained over the phone.

## 3. Results

### 3.1. Description of the Study Sample

Overall, 224 GERD patients were contacted, of those 149 (99 males [66.4%]) agreed to take part in the study, yielding a response rate of 65.5%. Among the participants, 67 (45.0%) patients had EE grade A, 32 (21.5%) had EE grade B, 41 (27.5%) had grade C, and 9 (6.0%) had esophagitis grade D. Most participants (n = 138, 92.6%) were Arabs and 11 (7.4%) were Jewish participants. The mean age at the baseline assessment was 44.6 years and at the follow-up it was 46.2 years. The mean number of schooling years of the participants was 11.7 years. Sixty participants (40.3%) were ever-smokers. About one third of the participants reported regular physical activity for 30 min at least once a week. Only 10.1% of the participants reported regular alcohol consumption. About one-third of the participants rated their health status as excellent, 38.3% reported having a very good health status, and 22.1% reported having a good health status (Table 1).

The percentage of males was lower in the A/B group than the C/D group: 61.6 vs. 76.0% (*p* = 0.05), as well as the percentage of married participants: 68.5 vs. 82.0% (*p* = 0.05). The mean number of schooling years was higher in participants with A/B esophagitis vs. C/D esophagitis: 12.1 vs. 10.8 years (*p* = 0.03). Patients with EE of grades C/D were more often overweight and obese when compared to patients with EE of grades A/B (*p* = 0.03). There was no significant difference between patients with EE grades A/B and those with grades C/D in the mean age at the baseline (*p* = 0.42) or at the follow-up assessments (*p* = 0.20), as well as for ethnicity (*p* = 0.28), smoking (*p* = 0.20), alcohol consumption (*p* = 0.38) and physical activity (*p* = 0.53) (Table 1).

### 3.2. Adherence to Recommendations and Utilization of Healthcare Services

All patients were treated with PPIs. The duration of PPI therapy in patients with EE grades A/B ranged between 1 and 48 months with a mean of 9.4 months (SD = 8.7), while the mean duration of PPI therapy in patients with EE grades C/D was 3.4 months (SD = 2.2). In this group, 14.1% of patients were treated with PPI for 1 month only, 21.2% of patients took PPIs for less than three months, 36.4% were treated PPIs for 3–12 months, and 28.3% took PPI for 13–48-months. Overall, 72 patients (72.7%) from the EE A/B group received a recommendation for PPI therapy at the index endoscopy. Only in 38.9% of these patients was the duration of PPI therapy stated at the endoscopy report. The recommendation for PPI therapy was documented among 27 patients (67.5%) with EE grades C/D. Among the 27 patients who received a recommendation for PPI therapy, only six patients (12.0%) received a recommendation for long period therapy. In this group, 24.0% of patients were treated with PPI for 1 month only, 64.0% of patients were treated with PPIs for up to three months, while 30.0% took PPIs for 4–12 months. A proportion of 28.3% of patients were treated with PPIs for a longer period (13–48-months). As mentioned, for patients with severe esophagitis (grades C/D), the recommendations include long term use of PPIs and a follow up gastroscopy after 2–3 months to exclude Barrett’s esophagus. Only three patients (6%) were adherent to the prolonged PPI treatment (more than 6 months).

Only 14 patients (14.2%) were non-adherent to the recommended treatment in the EE A/B group. Among patients from the C/D group, 21 patients (40.8%) were non-adherent to recommended treatment. The difference between both groups regarding adherence was significant (*p* < 0.001). No significant differences were found between patients with EE grades A/B and C/D in the type of PPIs (*p* = 0.14), dose of PPIs (*p* = 0.7), change in dietary habits (*p* = 0.85), or in following the referral to a gastroenterology clinic for follow up (*p* = 0.59), or a dietician clinic (Table 2).

### 3.3. Bivariate Analysis of Associations of Demogrpahics and Clinical Factors with Adherence

Patients with mild esophagitis in the group EE–A/B were more adherent to treatment when compared to patients with more severe esophagitis in the group EE–C/D (81.3 vs. 18.7%, *p* < 0.001). Moreover, the number of schooling years was associated with better adherence (*p* = 0.03). The symptom severity variables did not follow a normal distribution, therefore, categorical variables of symptoms severity were created (above median = 1, median and below = 0). To assess the association between symptoms’ severity and adherence we have used the Chi-squared test (dichotomic variables) and found no correlation between the regurgitation scale above median and adherence (*p* = 0.57), no correlation between the heartburn scale above median and adherence (*p* = 1.00), as well as between the dyspepsia scale above median and the adherence (*p* = 0.71). All other variables were not significantly associated with adherence (Table 3). Moreover, we have assessed the association between adherence and the existence of PPI therapy recommendation at the endoscopy report and found that 79.4% of the adherent patients received a PPI treatment recommendation at the endoscopy report, whereas among 51.4% of the non-adherent patients, no recommendation appeared at the endoscopy report (*p* = 0.001).

### 3.4. Multivariable Logistic Regression Models for Factors Associated with Adherence

Multivariable logistic regression models were performed to assess associations between adherence to treatment and esophagitis severity, while adjusting for potential confounders (Table 4). We selected the model with the best Nagelkerke R square (0.38). According to this model, the esophagitis severity was associated with adherence; more severe C/D esophagitis patients were less adherent when compared to the EE–A/B group (OR = 0.06; CI 95% 0.02–0.18, *p* < 0.01). Additionally, marital status was associated with adherence; the unmarried patients when compared to married ones were less adherent, (OR = 0.23; CI 95% 0.08–0.69, *p* < 0.001). The other variables were not significantly associated with adherence to treatment (Table 4).

Multivariable logistic regression models were performed to assess associations between adherence to treatment and symptoms’ severity, while adjusting for potential confounders (Supplementary Table S1). According to these models, no correlations were observed between symptoms severity (regurgitation, heartburn, and dyspepsia) and adherence to treatment.

## 4. Discussion

In the present study we evaluated the adherence to medical therapy and have assessed factors associated with adherence in patients with various degrees of erosive esophagitis. The main findings of this study are: patients with mild–moderate esophagitis (EE–A/B) were more adherent to medical therapy when compared to patients with more severe esophagitis (EE–C/D) and that unmarried patients were less adherent. Adherence to medical therapy is an important factor to control GERD symptoms and for the prevention of its complications. Poor adherence plays a key role in GERD management failure [12]. Generally, adherence is associated with many factors including, cultural, social, fear from chronic medication use, medications side effects, as well as factors related to patients such as awareness, understanding, education, financial state, and the relationship between healthcare provider and the patient [13,14]. Remarkably, the assessment of adherence to medical treatment is generally hindered by the absence of a standard tool or gold standard for this purpose. [12] A review paper that assessed the medical adherence/compliance of GERD patients has presented the various methods used to determine this as well as the wide spectrum of the reported compliance rates [15].

According to our study, patients with mild–moderate esophagitis (EE–A/B) were more adherent to treatment compared to patients with more severe esophagitis (EE–C/D). This might be the result of including follow-up endoscopy to the definition of adherence for patients with advanced EE, otherwise if focusing on medications only, the more severe patients had a much better adherence rates, but they were still smaller than the mild moderate esophagitis (EE–A/B) patients. An observational multi-center study from the Netherlands that included 4929 patients with GERD taking PPIs showed that 88% of patients were adherent to medical therapy as recommended by physicians [14]. Similar adherence rates were observed in another study conducted in the United States in which 84% of GERD patients were adherent to medical therapy. Other studies reported higher non-adherence rates of 30 [19] and 47% [20]. A review by Domingues et al. found that the level of education, patient–physician communication, co-morbidities, patient’s satisfaction, and advanced patient’s age were all associated with a higher level of adherence [15]. A study by Kamloz et al. revealed that the presence and severity of GERD symptoms and/or the presence of Barrett’s esophagus, increase patients’ adherence to PPI therapy [21]. Unlike these studies, our study found no significant associations between symptoms severity and adherence. Possible explanations for this discrepancy include the use of different tools to assess adherence and symptoms’ severity, different patients’ populations with different demographics and endoscopic findings, as well as the personal decision of patients to stop treatments after a short period, believing they are ineffective. Nonetheless, our study results are in concordance with other studies, where the GERD symptoms severity was not correlated with adherence to treatment [22,23].

We found that unmarried patients were less adherent to treatment recommendations. Marital status is generally an important factor affecting a patient’s approach to their health state and wellbeing, probably due to increased responsibility or commitment towards their families, or possibly, in some cases, compliance and adherence is encouraged and boosted by family members [24]. Previous reports on different medical conditions that studied of the relationship between marital status and medication adherence have produced inconsistent results [25,26,27,28].

Additionally, clear, detailed, comprehensive recommendations by physicians are crucial to patient’s understanding and the perception of their medical condition, leading to a better adherence and compliance [15]. For patients with severe esophagitis (grades C/D), the recommendations include the long-term use of PPIs and a follow up gastroscopy after 2–3 months to exclude Barrett’s esophagus. Overall, 72 patients (72.7%) from the EE–A/B group received a recommendation for PPI therapy at the index endoscopy. Only in 38.9% of these patients was the duration of PPI therapy stated at the endoscopy report. Notably, we found that the documentation of PPI treatment at the endoscopy reports improves adherence. Therefore, efforts should be invested to further improve endoscopy report quality [29]. Likewise, for the physician’s recommendations to change dietary habits and the referrals to gastroenterology clinic for follow or dietician clinic, all were insufficiently documented at the endoscopy reports, for both groups of patients.

The main limitations of our study are: (1) information on several variables was obtained through interviews with the participants; there might be some reporting bias on some variables such as smoking status and alcohol drinking (due to stigma in a conservative population). This might yield non-differential misclassification of these variables. Nonetheless, information on most medical variables was validated against patient’s medical electronic files. (2) Non-differential misclassification bias could occur with the estimation of the esophagitis grade between different gastroenterologists since variation between physicians might exist. (3) Our study included patients with erosive esophagitis while no NERD patients were included, narrowing the spectrum of GERD patients, which limits the generalizability of our findings to EE only. Moreover, the cohort is not representative to the general Israeli population since the majority of our patients were Arabs, with a special cultural and ethnical uniqueness. This is not expected to affect the external validity of results on the association of GERD EE grade with adherence to treatment. Nonetheless, the external validity regarding the adherence rates is limited to the population in the Nazareth area.

The main strength points of our study are: it’s prospective nature, the detailed data collection, and its validation against medical files.

To conclude, patients with mild–moderate esophagitis (EE–A/B) group were more adherent to medical therapy when compared to patients with more severe esophagitis (EE–C/D). Unmarried patients were less adherent.

## Figures and Tables

**Table 1:** Description of the study sample.

	Overall n = 149	EE Grades A/B (n = 99)	EE Grades C/D (n = 50)	*p* *
**Mean age at baseline (years), (SD)**	44.5 (15.1)	43.8 (14.4)	46.1 (16.4)	0.40
**Mean age at follow up (years), (SD)**	46.2 (14.9)	45.0 (14.4)	48.5 (15.9)	0.20
**Sex**				0.05
Male	99 (66.4%)	61 (61.6%)	38 (76.0%)	
Female	50 (36.6%)	38 (38.4%)	12 (24.0%)	
**Ethnicity**				0.28
Arabs	138 (92.6%)	93 (93.9%)	45 (90.0%)	
Jews	11 (7.4%)	6 (6.1%)	5 (10.0%)	
**BMI (kg/m^2^) ((continuous variable) (mean), (SD))**	28.4 (4.6)	28.0 (4.9)	29.0 (3.8)	0.23
**BMI (categorical)**	24 (24.2%)	5 (10.0%)		
**BMI 20–24 (normal)**	48 (48.4%)	25 (50.0%)		0.03
**BMI 25–29 (overweight)**	27 (27.2%)	20 (40.0%)		
**BMI ≥ 30 (obesity)**				
**Marital status**				0.05
Married	108 (72.5%)	68 (68.4%)	41 (82.0%)	
Not married (single, divorced, widow)	40 (27.5%)	31 (31.6%)	9 (18.0%)	
**Number of schooling, years, mean, (SD)**	11.7 (3.3)	12.1 (3.3)	10.8 (3.2)	0.03
**Smoking**				0.20
Ever smoker (past and current)	60 (40.3%)	37 (37.4%)	23 (46.0%)	
Never smoker	89 (59.7%)	62 (62.6%)	27 (64.0%)	
**Alcohol consumption**				0.38
Yes	15 (10.1%)	9 (9.1%)	6 (12.0%)	
No	102 (68.5%)	90 (90.9%)	44 (44.0%)	
**Physical activity for 30 min at least once a week**				0.53
Yes	47 (31.5%)	31 (31.3%)	16(32.0%)	
No	102 (68.5%)	68 (68.7%)	34 (68.0%)	

BMI: body mass index, SD: standard deviation, EE: erosive esophagitis. * *p* value was obtained by the Chi-squared test for categorical variables and the Mann–Whitney test for continuous variables.

**Table 2:** Comparison between patients with mild-moderate EE grades A/B and those with severe EE grades C/D in adherence to treatment and health services utilization.

	EE Grades A/B (n = 99)	EE Grades C/D (n = 50)	*p* *
**Adherence to treatment, yes**	85 (85.8%)	29 (59.2%)	**<0.001**
PPI therapy recommended at the endoscopy report	72 (72.7%)	27 (67.5%)	0.53
PPI therapy duration documented at the endoscopy report	28 (38.9%)	6 (12.0%)	0.12
**Type of PPI**			0.14
Nexium	35 (35.5%)	36 (72.0%)	
Lanton	24 (24.2%)	7 (14.0%)	
Omepradex	21 (21.2%)	6 (12.0%)	
Contraloc	1 (1.0%)	1 (2.0%)	
**Dose of PPI recommended**			0.71
20 mg	58 (58.6%)	27 (54.6%)	
30 mg	24 (24.2%)	7 (14.0%)	
40 mg	17 (17.2%)	16 (32.0%)	
**Dietary recommendation by physician**	37 (37.4%)	20 (40.0%)	0.85
**Did you change your dietary habits as recommended?**			0.85
Yes	32 (32.3%)	17 (34.0%)	
No	67 (67.7%)	33 (66.0%)	
**Did you change your diet as recommended**			0.50
Yes	17 (17.2%)	11 (22.0%)	
No	82 (82.8%)	39 (78.0%)	
**Visit to specialist in gastroenterology**			0.12
Yes	10 (10.1%)	10 (20.0%)	
No	89 (89.9%)	40 (80.0%)	
**Referral to a dietician**			0.73
Yes	6 (6.1%)	4 (8.0%)	
No	93 (93.9%)	46 (92.0%)	
**Visit to a dietician**			1.00
Yes	20 (20%)	12 (25.0%)	
No	80 (80.0%)	37 (74.0%)	
**Emergency room visit due to GERD symptoms**			0.38
Yes	17 (17.2%)	12 (24.0%)	
No	82 (82.8%)	38 (76.0%)	

EE: erosive esophagitis. * *p* value was obtained by the Chi-squared test for categorical variable.

**Table 3:** Bivariate analysis of associations of demographic and clinical factors with adherence.

	Adherence to Treatment (n = 107)	No Adherence to Treatment (n = 41)	*p* *
**Degree of esophagitis**			**<0.001**
EE–C/D	20 (18.7%)	29 (70.7%)	
EE–A/B	87 (81.3%)	12 (29.3%)	
**Sex**			0.84
Male	70 (65.4%)	28 (68.3%)	
Female	37 (34.6%)	13 (31.7%)	
**BMI (kg/m^2^) (categorical)**			0.87
BMI 20–24	22 (20.8%)	7 (17.1%)	
BMI 25–29	51 (48.1%)	21 (51.2%)	
BMI ≥ 30	33 (31.1%)	13 (31.7%)	
**Marital status**			0.30
Unmarried	26 (74.5%)	14 (34.1%)	
**Symptoms severity (RDQ)**			0.57
Regurgitation scale above median	43(40.2%)	19 (46.3%)	
Heartburn scale above median	49(45.8%)	19 (46.3%)	1.00
Dyspepsia scale above median	43 (40.2%)	18 (43.9%)	0.71
**PPI treatment at endoscopy report**	99 (79.4%)	19 (51.4%)	0.001
**Married**	80 (75.5%)	27 (65.9%)	
**BMI (kg/m^2^) ((continuous) (mean), (SD))**	28.23 (4.2)	28.93 (5.3)	0.82
**Age, mean, (SD)**	43.55 (14.1)	47.29 (16.7)	0.13
**Number of schooling years mean, (SD)**	12.02 (3.2)	10.71 (3.4)	**0.03**
**Charlson’s index, mean, (SD)**	1.41 (2.4)	2.04 (2.7)	0.26

BMI: body mass index, SD: standard deviation, EE: erosive esophagitis, RDQ: reflux disease questionnaire. * *p* value was obtained by the Chi-squared test for categorical variable and the Student’s *t*-test for continuous variables.

**Table 4:** Multivariable logistic regression model for factors associated with adherence to treatment and esophagitis severity among GERD patients.

Variable	OR (95% CI)	*p*
Marital status		**<0.001**
(Unmarried vs. married)	0.23 (0.08–0.69)	
Age (years)	0.97 (0.94–1.01)	0.24
Sex		0.62
(Female vs. male)	0.78 (0.29–2.06)	
Schooling years	1.04 (0.90–1.21)	0.53
Esophagitis severity		**<0.001**
(C/D vs. A/B)	0.06 (0.02–0.17)	
**BMI (kg/m^2^) (categorical)**		
BMI 20–24	Reference	
BMI 25–29	1.52 (0.43–5.36)	0.54
BMI ≥ 30	1.91 (0.49–7.40)	0.34

Nagelkerke model = 0.38, CI: confidence interval; OR: odds ratio, BMI: body mass index.

## Data Availability

The data are found at the gastroenterology department at EMMS Nazareth Hospital, Nazareth, Israel. Individual level data can’t be publicly available due to legal and ethical restrictions.

## Supplementary Materials

The following supporting information can be downloaded at: https://www.mdpi.com/article/10.3390/jcm11113196/s1, Table S1: Multivariable logistic regression model for factors associated with adherence to treatment and symptoms severity (regurgitation); Table S2: Multivariable logistic regression model for factors associated with adherence to treatment and symptoms severity (heartburn); Table S3: Multivariable logistic regression model for factors associated with adherence to treatment and symptoms severity (dyspepsia).

## Abbreviations

BMI	Body Mass Index
CI	Confidence Interval
EE	Erosive Esophagitis
GERD	Gastro-esophageal Reflux Disease
OR	Odds Ratio
PPI	Proton pump inhibitors
RDQ	Reflux Disease Questionnaire
SD	Standard Deviation
