# Supplementary material for: Comparison in Adherence to Treatment between Patients with Mild–Moderate and Severe Reflux Esophagitis: A Prospective Study
Source: J Clin Med. 2022 Jun 3;11(11):3196. doi: 10.3390/jcm11113196 (PMC9181805; doi:10.3390/jcm11113196)
Supplement: Supplementary file 1 [file jcm-11-03196-s001.zip › jcm-1719740-supplementary.pdf]

Journal of
Clinical Medicine

MDPI

Supplementary Tables

Table S1. Multivariable logistic regression model for factors associated with adherence to treatment and symptoms severity (regurgitation).

Variable	OR (95% CI)	*p*
adherence	1.30 (0.54–3.15)	0.55
Age (years)	0.97 (0.94–1.01)	0.06
Sex (Female vs. male)	1.27 (0.61–2.63)	0.51
Schooling years	0.94 (0.84–1.21)	0.39
Esophagitis severity (C/D vs. A/B)	2.38 (1.02–5.56)	**0.04**
Charlson index	1.21 (1.00–1.47)	**0.04**

CI: confidence interval; OR: odds ratio.

Table S2. Multivariable logistic regression model for factors associated with adherence to treatment and symptoms severity (heartburn).

Variable	OR (95% CI)	*p*
adherence	1.62 (0.66–3.99)	0.28
Age (years)	0.97 (0.93–0.99)	**0.04**
Sex (Female vs. male)	2.22 (1.07–4.61)	**0.03**
Schooling years	0.94 (0.84–1.06)	0.36
Esophagitis severity (C/D vs. A/B)	2.77 (1.17–6.56)	**0.02**
Charlson index	1.09 (0.90–1.31)	0.34

CI: confidence interval; OR: odds ratio.

Table S3. Multivariable logistic regression model for factors associated with adherence to treatment and symptoms severity (dyspepsia).

Variable	OR (95% CI)	*p*
adherence	1.31 (0.53–3.25)	0.55
Age (years)	0.98 (0.94–1.01)	0.22
Sex (Female vs. male)	3.24 (1.54–6.801)	**<0.001**
Schooling years	0.89 (0.79–1.01)	0.09
Esophagitis severity (C/D vs. A/B)	1.98 (0.85–4.73)	0.12
Charlson index	1.07 (0.89–1.29)	0.42

CI: confidence interval; OR: odds ratio.
